# Traditional Chinese medicine for treating aplastic anemia

**DOI:** 10.3389/jpps.2023.11863

**Published:** 2023-11-13

**Authors:** Jing Guan, YiHui Zhao, Ting Wang, Rong Fu

**Affiliations:** Department of Hematology, Tianjin Medical University General Hospital, Tianjin, China

**Keywords:** treatment, aplastic anemia, traditional Chinese medicine, mechanism, Chinese materia medica

## Abstract

Aplastic anemia (AA) is a bone marrow failure disease caused by T cell hyperfunction. Although the overall response rate has been improved by immunosuppressive therapy (IST) plus Eltrombopag, 30% of patients have either no response or relapse. We therefore attempted to find other ways to improve the outcomes of AA patients. Traditional Chinese medicine has the advantages of low cost, reasonable effects, and few side effects. More and more clinical studies have confirmed that traditional Chinese medicine has a beneficial role in treating AA patients. This article reviews the potential mechanism of traditional Chinese medicine or its active ingredients in the treatment of AA. These include improving the bone marrow microenvironment, regulating immunity, and affecting the fate of hematopoietic stem cells. This provides useful information for further treatment of AA with integration of traditional Chinese and Western medicine and the development of new treatment strategies.

## Introduction

Aplastic anemia (AA) is a bone marrow failure disease, characterized by T cell hyperfunction attacking bone marrow hematopoietic stem/progenitor cells (HSC/HPC), resulting in bone marrow failure and peripheral blood cytopenia [1]. In traditional Chinese medicine (TCM), according to the clinical symptoms, AA belongs mainly to the category of “myeloid fatigue,” “blood disease” and “consumptive disease,” which are caused by spleen and kidney deficiency, blood collateral damage, and blood stasis. The corresponding treatment measures should be to strengthen the spleen and kidney, purging fire and cooling blood, and promoting blood circulation to remove blood stasis [2]. In recent years, the related research of AA treated with Chinese medicine has been used widely at home and abroad. Studies have shown that TCM plays a therapeutic role in various ways and shows reasonable efficacy in the field of AA treatment. In this review, we mainly discuss the clinical application of TCM in AA, paying special attention to the potential mechanism of TCM application.

## Clinical application of traditional Chinese medicine in aplastic anemia

For AA patients who can undergo hematopoietic stem cell transplantation (Allo-HSCT), Allo-HSCT treatment can be performed. However, the role of Allo-HSCT is limited by age, matching, and many other factors such as graft-versus-host disease. Immunosuppressive therapy (IST) has been successfully used in the treatment of AA patients, but there are still 20%–40% who have a low response to IST [3]. Chinese medicine has achieved remarkable results in the treatment of AA, which provides useful ideas and methods for the clinical treatment of AA. Combining traditional Chinese and Western medicine with its advantages is an important area in the treatment of AA. It has been reported that the total effective rate of TCM combined with IST in treating SAA was 85.9%, the median starting time was 4.5 months, and there were fewer adverse reactions [4]. A total of 111 CAA patients in many centers were divided randomly into Chinese medicine groups of tonifying kidney (KA), tonifying kidney and replenishing qi (KQ) and tonifying kidney and activating blood (KC), and were grouped into either normal or positive control groups. All the patients were treated with cyclosporine (CsA) and androgen. After 6 months of treatment, the study showed that the total effective rate of the KA, KQ, and KC groups was higher than that of the positive control group, while the symptoms scores of TCM of the KQ and KC groups were significantly lower than that of the positive control group [5]. Similarly, a prospective, randomized, double-blind, placebo-controlled, multicenter clinical study compared the clinical results of 567 AA patients treated with Bushen Shengxue and Yiqi Yangxue Granules combined with Western medicine. The results showed that compared with the placebo group, the combination of traditional Chinese and Western medicine promoted the recovery of the hemogram, reduced blood transfusion dependence, and improved the quality of life [6]. Zhu CT et al also conducted a meta-analysis of clinical controlled trials and showed that androgen combined with an astragalus injection increased peripheral blood cells and improved hematopoiesis, with the total effective rate increasing by 50% compared with that observed with androgen alone [7]. In addition, a study showed that a kidney-reinforcing, blood-activating and stasis-removing recipe combined with allo-HSCT improved the implantation of AA HSCs, reduced the risk of transplant failure, and increased the 5 years overall survival (OS) rate to 81.8% [8]. However, the small sample size and randomness limit of the clinical trials of Chinese medicine combined with Western medicine in the treatment of AA, make the need for a well-designed large sample study of great significance.

## The mechanism of Chinese medicine in treating aplastic anemia

### The role of TCM treatment in promoting the hematopoietic microenvironment of AA bone marrow

#### TCM treatment promotes homing of AA hematopoietic stem/progenitor cells

Bone marrow mesenchymal stem cells (MSCs), as an important part of the hematopoietic microenvironment, can differentiate into osteoblasts, chondrocytes, and adipocytes, and secrete a large number of cytokines and growth factors, thereby affecting hematopoietic function [9]. Bone marrow MSCs secrete chemokines that regulate the adhesion, expansion, migration and homing of HSCs. VCAM-1, VLA-4, and CD44 help HSCs home to the niche of the bone marrow microenvironment by promoting the interaction between HSCs and bone marrow MSCs, which is conducive to their growth and development. Studies have shown that the expression levels of CD106, CD49d, CD31, and CD44 in bone marrow MSCs of patients with chronic aplastic anemia (CAA) are significantly lower than those of healthy donors. After treatment with a kidney-reinforcing recipe (KRR) including Radix Rehmanniae Praeparata, blood-activating and stasis-removing recipe (BASRR) including Radix et Rhizoma Salviae Miltiorrhizae and kidney-reinforcing, blood-activating and stasis-removing recipe (KRBASRR) including above all, the expression of CD106, CD49d, CD31 and CD44 of MSCs was increased in varying degrees. Moreover, the expression of adhesion molecules in bone marrow MSCs by the KRR combined with the BASRR group was significantly higher than that of KRR group and BASRR group alone. The results showed that Chinese medicine promoted the homing and development of HSC by increasing the expression of adhesion molecules in bone marrow stromal cells of AA patients, thereby promoting homing and development of HSCs [10]. However, the specific mechanism requires further study. In addition, the VLA6/laminin receptor (CD49F) is expressed widely in artificial blood stem cells and progenitor cells, and plays an important role in HSC homing [11]. Busui Shengxue Granules are a TCM that contain the active ingredients of Radix Rehmanniae Preparata, Ginseng Radix et Rhizoma Rubra and Caulis Spatholobi. This medicine has the functions of Tonifying Kidney Yang, generating marrow, and nourishing blood. The results of studies have shown that compared with a control group, the expression of VLA-6/CD49F in bone marrow mononuclear cells of CAA patients in the Busui Shengxue Granule treatment group was increased significantly, and that the expression of ligand LN in peripheral serum returned to normal levels, which improved the migration and homing ability of HSCs [12]. In addition, peripheral blood Ln expression levels in CAA patients can predict the therapeutic effect of Bushui Shengxue Granules. This study shows that Bushui Shengxue Granules have a better therapeutic effect on patients with kidney yang deficiency than those with kidney yin deficiency. Fibronectin (FN) is a kind of cell adhesion glycoprotein, which mediates the interaction between cells and cells and matrix by binding with cell surface receptors, thereby affecting cell adhesion, migration, proliferation, and differentiation [13]. A study found that Busui Shengxue Granules can also restore the abnormally elevated FN level in CAA, enhance the adhesion of bone marrow hematopoietic cells and stromal cells, resulting in restoration of bone marrow hematopoiesis [14]. However, abnormal expression of adhesion molecules on other cells in bone marrow microbiota is also involved in the pathogenesis of AA. It is necessary to further determine whether there are multi-target effects of drugs and interactions between targets to provide more scientific basis for drug application.

#### Improving the differentiation of bone marrow mesenchymal stem cells

Bone marrow MSCs of AA patients tend to differentiate into adipocytes [15]. Excessive adipogenesis results in numerous adipocytes occupying the bone marrow space and damaging hematopoiesis [16]. The bone marrow pathology of AA model mice showed that the number of blood sinuses was reduced significantly by the replacement of hyperplastic adipose tissue. In some adipose tissue stroma, reticular cells proliferated, accompanied by infiltration of a large number of lymphocytes and plasma cells. Compared with the AA model group, the group treated with Danggui Buxue Tang (DBT,a Chinese herbal decoction) had more blood sinuses and less adipose tissue, indicating that this treatment improved the differentiation of bone marrow MSCs, regulated the bone marrow microenvironment, and promoted hematopoiesis [17]. The specific mechanism remains to be further explored. Rehmannia glutinosa polysaccharide (RGP) is a bioactive compound present in the roots of Rehmannia glutinosa, which can promote the osteogenic differentiation of bone marrow mesenchymal stem cells. One study confirmed that RGP alone or combined with CsA can decrease adipocytes and promote hematopoiesis on AA mice. In particular, the pathological state of bone marrow in the combined group was close to that of the normal group. RGP can also regulate inflammatory cytokines in the bone marrow by inhibiting the activation of the HIF-1α/NF-κB pathway, thereby improving bone marrow microarray [18].

#### Regulation the aging and proliferation of bone marrow mesenchymal stem cells

Compared with healthy people’s bone marrow MSCs, the proliferation of these cells in AA patients is decreased. At the same time, the aging of bone marrow MSCs in the hematopoietic microenvironment is considered to be another important cause of AA [19]. Ginseng is regarded as the king of oriental herbs and has two functions of invigorating qi and nourishing blood. Ginsenoside Rg1, a steroid saponin rich in ginseng, is the main active component of ginseng. There is evidence that Rg1 significantly promotes the proliferation of bone marrow MSCs in a dose-dependent manner by delaying the aging of these cells, restoring the level of hematopoietic factors, and improving the proliferation ability of BMNC [20]. In addition, Rg1 has an indirect effect on BMSCs by inhibiting inflammatory response and oxidative damage. Rg1 has estrogen-like properties, with results showing that Rg1 activates the expression of ERα in BMSC through estrogen, a downstream signal molecule, and promotes the proliferation of bone marrow stromal cells [21]. Rg1 also reduces the expression of P53 and P16 in aging BMSCs, reduces the competitive inhibition of CDK2 and CDK4, and increases the specific binding of cyclinD/CDK4 and cyclinE/CDK2, thereby alleviating the aging of the hematopoietic microenvironment and maintaining the normal proliferation of hematopoietic cells [20]. DBT, which consists of Radix Angelicae Sinensis, Radix Astragali seu Hedysari, and Rhizoma Coptidis is used widely in TCM to treat anemia and inflammation. It has been reported that DBT increases the number of BMSCs through the classical focal adhesion signaling pathway, up-regulates the expression of integrinα, increases the expression and phosphorylation of cytoplasmic protein tyrosine kinase FAK, induces the phosphorylation of pile protein, regulates the activity of focal adhesion protein, and recruits cytoskeleton and F- actin. These actions increase the number of BMSCs and promote their adhesion and migration, which indicates that these cells are one of the target cells of DBT to promote hematopoiesis [22]. The specific active ingredients of DBT can be studied in the future using bone marrow stromal cells as a cell model.

### Immune regulation of AA by treatment with Chinese medicine

#### Regulation of immune cells and immune factors

Immunological pathogenesis is considered as the core mechanism of AA. Abnormal cellular immunity destroys HSCs, leading to hematopoietic suppression and pancytopenia. An attack of CD8^+^ cytotoxic T cells (CTL) on HSCs is the most direct cause of AA. At the same time, there are many immune disorders in AA patients, such as the amplification and activation of Th1 and Th17, and a decrease in the number and function of Th2 and Tregs. The corresponding disorder of inflammatory factors, such as an abnormal increase of IFNγ, IL-2, TNFα, and IL-12 and abnormal expression of transcription factors such as an increase of T-bet and STAT1 which guide Th1 cell differentiation, increased expression of the specific transcription factor RORγt required by Th17 cells, and a decrease in transcription factor, GATA-3 which promotes Th2 cell differentiation [23]. CD28 is a costimulatory molecule on T lymphocytes, which activates T cells and delays their apoptosis. It was found that the expression levels of the co-stimulatory molecules, CD28 and CD8^+^CD28^+^, in CAA patients with kidney-yin deficiency were significantly higher than those in patients with kidney-yang deficiency. This indicated that the degree of immune disorder in patients with kidney-yin deficiency was significantly higher than that in patients with kidney-yang deficiency [24]. Many traditional Chinese medicines used to treat AA by regulating immunity. Sodium copper chlorophyll (SCC) extracted from silkworm excrement can restore hematopoiesis like cyclosporine (CsA), but the mechanism is different. Compared with the AA model group, the CsA group had significant CD8 cells and TNF-α and IFN-γ levels, while the high-dose SCC group had significant CD4 cells and Treg cells, which may all be related to the Fas expression of BM-MSCs [25]. The relevance of its effect and the concentration dependence of SCC need to be further confirmed. The study identified 18 active components of derivatives of DBT (DGBX) through HPLC-MS and found that multiple components of DGBX reduced the expression of key molecules in the JAK/STAT signaling pathway, reduced the production of inflammatory factors IFNγ, IL-2, IL-6, and IL-17 and also the number of Th1 and Th17 cells, increased the expression of GATA-3, promoted Th2 cell differentiation, and improved the abnormal immune response of a AA mouse model [26]. HPLC-MS conducts pharmacological studies by identifying active compounds in Chinese herbal formulas, making it possible to study the synergistic therapeutic effects of multiple targets. Radix Astragali seu Hedysari, as a biological regulator for enhancing immunity, promotes hematopoiesis by increasing the CD4/CD8 ratio and decreases negative regulatory factors such as IL-2 and TNF-α [27]. The TCR’s helper co-receptor signaling lymphocyte activating molecule (SLAM) and its downstream signaling molecule SLAM-related protein (SAP) inhibit the expression of IFNγ [28]. It has been reported that DGBX significantly activates the SLAM-SAP signal of AA mice to down-regulate the level of IFNγ and regulate the STAT/JAK/IRF-1 pathway, thus improving the immune status of AA [29]. Its hematopoiesis-promoting and immunosuppressive effects are comparable to or even greater than those of CsA. The panaxadiol saponin component (PDS-C) isolated from total ginsenoside is used widely in the treatment of pancytopenia. It was shown that PDS-C corrected the imbalance of T cell subsets by down-regulating T-bet and up-regulating the expression of GATA-3 and FoxP3 [30]. Dioscorea saponin (DNS) found in the rhizome of Pangolin, which contains a variety of active ingredients of steroidal saponins, can promote hematopoietic recovery in AA mice. The mechanism may be to restore Th17/Treg by regulating the Notch/RBPJj/FOXP3/RORγt pathway [31]. Interestingly, this study proposed that the Notch/RBPJj/Foxp3/ROCγt axis may be involved in the pathogenesis of AA and can be further explored. The PI3K/AKT and STAT3-RORγT signaling pathways regulate the expression of Foxp3, a functional molecule of Treg cells involved in their differentiation [32]. The therapeutic effect of Lulongzaisheng decoction on AA is exerted mainly by inhibiting STAT3 phosphorylation, increasing Foxp3 expression of CD4^+^CD25+Tregs cells, and increasing the number of CD4^+^CD25+Treg cells in AA mice [33]. This study provides a theoretical basis for the treatment of AA with Lulong Zaisheng decoction. In CAA patients treated with the Shengxue Mixture, the ratio of Th1 cells decreased, with the ratio of Th1/Th2 being close to normal, which may be related to inhibition of abnormal activation of IFN-γ/T-bet and the IL-12/STAT4 pathway [34]. In addition, the expression level of serum tyrosine kinase −3 ligand in CAA patients was reported to decrease after treatment with Busui Shengxue Granules, thereby alleviating suppression of the bone marrow mediated by abnormal immune function [14].

#### Regulation of amino acid metabolism

Amino acids support the proliferation and function of immune cells by maintaining the redox balance, modifying DNA and histones, and promoting nucleotide synthesis [35]. The Compound Shenlu Granules (SLG) is a classic kidney-tonifying and blood-nourishing preparation for treating AA patients with the kidney-yang deficiency syndrome. Many of its active ingredients have immunomodulatory and hematopoietic promoting effects. Metabonomic analysis showed that SLG improves AA immune status by affecting amino acid metabolism, such as increasing tryptophan and phenylalanine content [36]. The sample size included in the study is relatively small, but it supports the positive role of metabolomics in evaluating the effectiveness and mechanism of action of treating AA. The compound is used in combination with Radix Astragali seu Hedysari and Flos Carthami to treat AA. It has reported in blood deficiency mice that Chinese medicine treatment improved their abnormal metabolism and regulated the metabolic pattern, including phenylalanine, tyrosine, tryptophan, valine, leucine, and isoleucine biosynthesis, and tryptophan and tyrosine metabolism [37].

#### Effect on T cell cloning

Patients with AA have an abnormal amplification of the β subfamily of T cell receptor variable region, which mediates immune hematopoietic inhibition in patients with chronic disease [38]. The Shengxue Mixture is composed mainly of Radix Astragali seu Hedysari, Radix Codonopsis, Radix Rehmanniae Recens, and Radix et Rhizoma Notoginseng. It is a compound Chinese medicine preparation and functions to invigorate the spleen, tonify the kidney, and promote blood circulation. It has been proved to be effective clinically in treating CAA for many years. Through RT- PCR and gene scanning, it was found that the Shengxue Mixture reduced the gene expression of the T cell receptor β chain variable region (TCR Vβ) subfamily, which indicated that the Mixture improved the immune-mediated injury of AA by affecting T cell cloning [39].

### The effect of TCM treatment on HSC

#### Inhibition of HSC apoptosis and promotion of HSC proliferation

AA is characterized by an increase in HSC apoptosis [15]. Apoptosis of AA stem cells is mediated mainly by Fas/FasL, and other apoptosis-related molecules such as Bax, Bcl-2, and NFκB. P53 is also involved. It has been shown that DGBX inhibits the apoptosis of bone marrow cells by affecting the activation of the Fas-dependent pathway [28]. Angelica sinesis polysaccharide (ASP) is a water-soluble polysaccharide, composed of glucuronic acid, glucose, arabinose, and galactose. It is one of the main active components extracted from Radix Angelicae Sinensis. Compared with the AA model group, ASP treatment significantly reduces the apoptosis rate and significantly restores the level of bone marrow HSCs. The mechanism of this reduction may involve the inactivation of Bax, caspase-9, caspase-3, ROS, and the p38/MAPK signaling pathways [40]. The role of Angelica in promoting hematopoiesis is also related to the interaction of SYK, JAK2, and ITK [41]. The analysis of biological interactions through interactive network data warehouse can help predict the therapeutic mechanism of traditional Chinese medicine, which is beneficial to further biomedical research. SCF, a hematopoietic stimulator, binds to the tyrosine kinase receptor c-kit and induces the proliferation and differentiation of hematopoietic stem/progenitor cells. The Bushen Shengxue Jiedu Formula, that includes the Danggui Buxue and Guilu Erxian Jiao Decoction, has been used in the clinical treatment of acute and chronic AA, and has achieved good therapeutic effects. These prescriptions promote the proliferation and differentiation of HSC by activating the SCF/c-kit signaling pathway [42], and also promote the differentiation and maturation of erythrocytes by activating the EPO/EpoR signaling pathway [43]. Fuzhengyangying Granules up-regulate the expression of Bcl-2 and play a role in the treatment of AA [44]. Panax notoginseng (PNS) is a type of high value Chinese herbal medicine, mainly in two forms, raw and steamed Panax notoginseng. Raw Panax notoginseng is used to treat inflammation and pain, while steamed Panax notoginseng (SPN) is used as a “blood tonic” to reduce anemia and enhance the body’s immunity. It has been shown that SPN supports hematopoiesis by upregulating the expression of EPO, EpoR, TPO, c-Mpl, GM-CSF, and GATA-1, and the anti-apoptotic proteins, Bcl-2 and Bcl XL [45], providing the possibility for SPN to be used in the treatment of bone marrow failure diseases such as AA. At the same time, SPN down-regulates the expression of Bax and inhibits apoptosis of HSC [46]. PNS also improves bone marrow suppression in AA by inhibiting the expression of Daxx and Fas protein. In addition, it increases expression of NF-κB and C-Rel protein to promote hematopoietic cell proliferation [47]. In addition, DNS can also regulate the expression of key proteins of the Fas pathway and inhibit bone marrow cell apoptosis in AA mice [48]. However, the specific mechanism of how DNS regulates the differentiation of CD34^+^ cells into various blood cell lineages in the bone marrow is unclear and deserves further exploration. Tetramethylpyrazine is an active alkaloid extracted from Ligusticum chuanxiong Hort and has been shown to improve the microcirculation by reducing oxidative stress. There is evidence that Ligustrazine reduces the expression of Fas in mice with immune-mediated bone marrow failure, thereby reducing apoptosis of HSC mediated by Fas/FasL [49]. Ligustrazine may be a potential new treatment for myelosuppressive pancytosis. As an adjuvant therapy for CAA, the Bushen Huoxue Tongluo recipe competitively inhibits Fas and blocks HSC apoptosis by increasing sFAS levels [50].

#### Increasing the number and improving the function of mitochondria in HSC

Acquired deletions and mutations of mitochondrial DNA result in mitochondrial dysfunction and blockage of energy supplies, leading to bone marrow failure in AA. It was demostrated that bone marrow mononuclear cells (BMMNC) of AA mice have a thinner mitochondrial membrane, reduced number, shortened cristae, abnormal shape, and decreased mitochondrial DNA content [51]. It has also been reported that Rg1 increased the number of mitochondria in BMMNC and the levels of ATP/ADP, reduced the level of mitochondrial apoptosis, and restored the energy supply of HSC in AA [52].

#### Regulation of gene expression of stem cells and improving colony forming ability of stem cells

The colony forming ability of HSC/HPC in AA is decreased. There are many abnormal genes expressed in AA stem cells, including apoptosis-related genes, cytokine genes, and signal transduction regulatory genes [53]. It has been reported that a PDS-C treatment group had increased colony formation of erythroid, myeloid, and megakaryocyte progenitor cells, up-regulated expression of GATA-1, GATA-2, c-fos, c-Jun and NF-E2, and increased protein expression of MAPK signaling pathway to restore hematopoiesis [54].

#### Inhibition of aging in HSC

Inherited and acquired AA is associated with decreased telomerase activity and shortened telomeres due to a telomerase reverse transcriptase (TERT) gene mutation, which ultimately leads to aging of HSC [15]. Telomerase inhibition activates the expression of P53, drives the expression of cyclin-dependent kinase (CDK) inhibitors such as P21, and induces cell senescence [55]. Studies have shown that ASP promotes HSC proliferation and inhibits HSC aging by activating telomerase, increasing telomere length and the CDK2/CyclinE complex, decreasing the expression of P53 protein, promoting Rb protein phosphorylation, and releasing transcription factor E2F [56].

## Discussion

**FIGURE 1:**
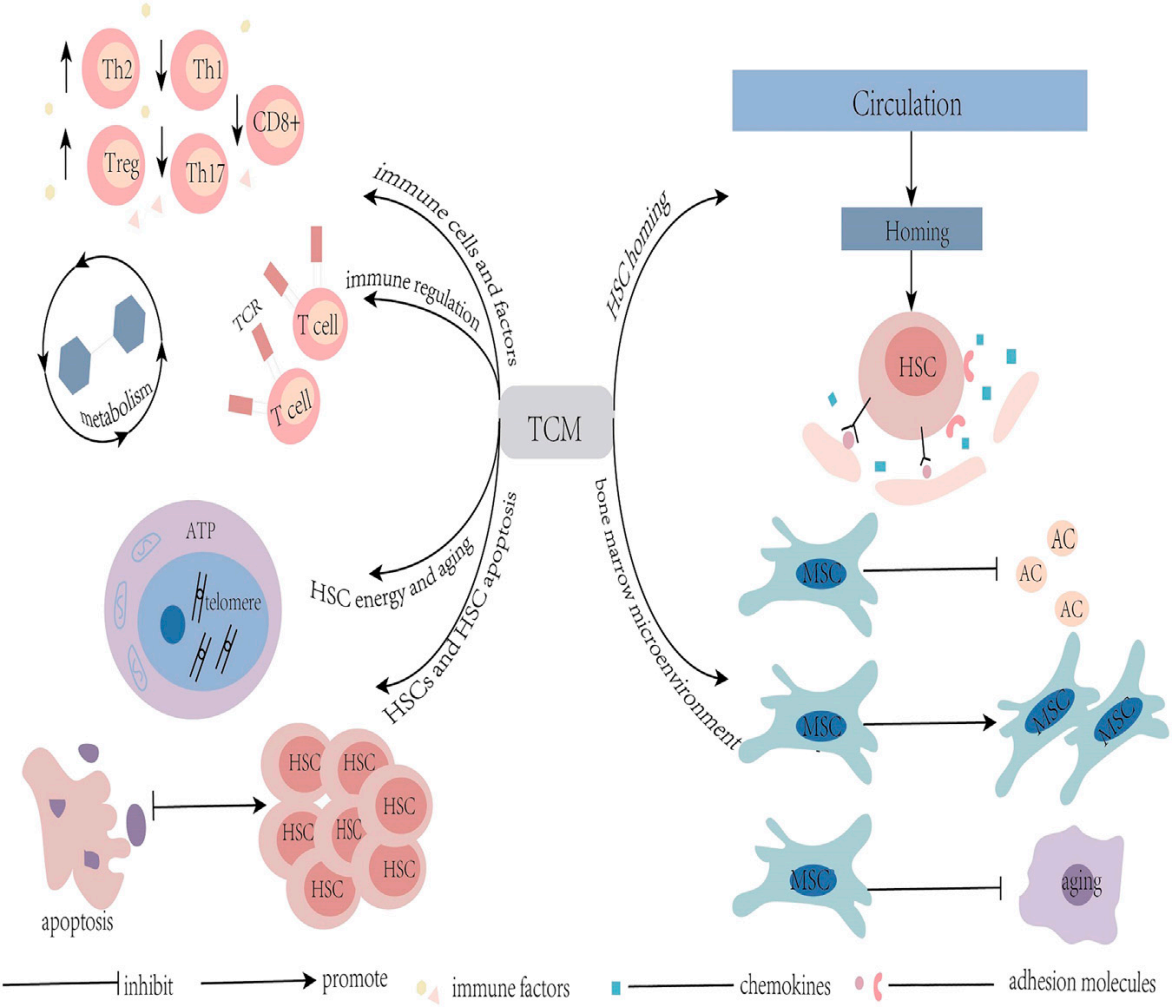
Graphical Abstract: Mechanisms of TCM in the treatment of aplastic anemia; TCM: traditional Chinese medicine; HSC: hemopoietic stem cells; MSC: mesenchymal stem cells; AC: adipose cells.

TCM can play a role in treating AA by improving the hematopoietic microenvironment of the bone marrow, regulating immunity, promoting proliferation and differentiation of HSC, and inhibiting aging and apoptosis of HSC (Figure 1). TCM can be used as an effective supplement to Western medicine in treating AA. In addition, TCM effectively reduces the toxic and side effects of Western medicine, and improves the treatment efficiency and overall survival rate of patients. The development of cytomics, proteomics, and metabolomics has contributed to a more comprehensive understanding of the underlying mechanisms of TCM treatment for AA and the development of new therapeutic strategies. However, in reality, due to uncertainty regarding the effective ingredients of TCM and an imbalance of the purity of the effective ingredients of herbal medicine, it is difficult to judge the pharmacokinetics, safety, and effectiveness of TCM. Clinical popularization is therefore restricted. Once this barrier is overcome, the combination of Chinese and Western medicine is expected to achieve better results in the treatment of AA.

## Abbreviations

AA, aplastic anemia; IST, immunosuppressive therapy; HSC/HPC, hematopoietic stem/progenitor cells; TCM, traditional Chinese medicine; KA, tonifying kidney; KC, tonifying kidney and activating blood; KQ, tonifying kidney and replenishing qi; CsA, cyclosporine; OS, overall survival; MSC, marrow mesenchymal stem cells; KRR, kidney-reinforcing recipe; BASRR, blood-activating and stasis-removing recipe; KRBASRR, kidney-reinforcing, blood-activating and stasis-removing recipe; CD49F, laminin receptor; FN, Fibronectin; DBT, Danggui Buxue Tang; CTL, CD8^+^ cytotoxic T cells; SLAM, signaling lymphocyte activating molecule; SAP, SLAM-related protein; PDS-C, panaxadiol saponin component; SLG, Shenlu Granules; TCR Vβ, T cell receptor β chain variable region; ASP, Angelica sinesis polysaccharide; PNS, Panax notoginseng; SPN, steamed Panax notoginseng; BMMNC, bone marrow mononuclear cells; TERT, telomerase reverse transcriptase; CDK, cyclin-dependent kinase.
